# Wnt Signaling Through Nitric Oxide Synthase Promotes the Formation of Multi-Innervated Spines

**DOI:** 10.3389/fnsyn.2020.575863

**Published:** 2020-09-04

**Authors:** Faye McLeod, Kieran Boyle, Aude Marzo, Nuria Martin-Flores, Thaw Zin Moe, Ernest Palomer, Alasdair J. Gibb, Patricia C. Salinas

**Affiliations:** ^1^Department of Cell and Developmental Biology, University College London, London, United Kingdom; ^2^Department of Neuroscience, Physiology and Pharmacology, University College London, London, United Kingdom

**Keywords:** multi-innervated spines, structural plasticity, LTP, Wnt signaling, nitric oxide

## Abstract

Structural plasticity of synapses correlates with changes in synaptic strength. Dynamic modifications in dendritic spine number and size are crucial for long-term potentiation (LTP), the cellular correlate of learning and memory. Recent studies have suggested the generation of multi-innervated spines (MIS), in the form of several excitatory presynaptic inputs onto one spine, are crucial for hippocampal memory storage. However, little is known about the molecular mechanisms underlying MIS formation and their contribution to LTP. Using 3D enhanced resolution confocal images, we examined the contribution of Wnt synaptic modulators in MIS formation in the context of LTP. We show that blockage of endogenous Wnts with specific Wnt antagonists supresses the formation of MIS upon chemical LTP induction in cultured hippocampal neurons. Gain- and loss-of-function studies demonstrate that Wnt7a signaling promotes MIS formation through the postsynaptic Wnt scaffold protein Disheveled 1 (Dvl1) by stimulating neuronal nitric oxide (NO) synthase (nNOS). Subsequently, NO activates soluble guanylyl cyclase (sGC) to increase MIS formation. Consistently, we observed an enhanced frequency and amplitude of excitatory postsynaptic currents. Collectively, our findings identify a unique role for Wnt secreted proteins through nNOS/NO/sGC signaling to modulate MIS formation during LTP.

## Introduction

Neuronal activity plays a crucial role in the establishment and refinement of neuronal networks (West and Greenberg, 2011). Activity induces functional and structural changes at the synapse through a process known as synaptic plasticity. In the mammalian brain, one of the main forms of synaptic plasticity is long-term potentiation (LTP), thought to underlie experience-dependent learning and memory. It is well established that activation of N-methyl-D-aspartate receptors (NMDARs) can induce LTP through the CaMKII cascade resulting in increased dendritic spine size and synaptic strength (Malenka and Bear, 2004; Herring and Nicoll, 2016). Interestingly, LTP induction also increases the formation of spines innervated by two or more presynaptic boutons, called multi-innervated spines (MIS) (Nikonenko et al., 2003). However, little is known about mechanisms controlling MIS formation during LTP induction.

Recent studies show that MIS generation facilitates long-term memory (Radwanska et al., 2011; Giese et al., 2015; Aziz et al., 2019). In the hippocampus, multi-synaptic filopodia/atypical spines are common immature synaptic structures during early development but the formation of MIS is rare, representing less than 1% of the total excitatory synapses under basal conditions in the adult (Fiala et al., 1998; Petrak et al., 2005; Radwanska et al., 2011). In organotypic hippocampal cultures, activation of postsynaptic NMDARs or expression of the postsynaptic scaffold proteins PSD-95 or SAP-97 induces the formation of MIS (Nikonenko et al., 2003, 2008; Poglia et al., 2011). Importantly, the generation of MIS requires nitric oxide (NO) signaling. PSD-95 binds to neuronal NO synthase (nNOS), resulting in NO messenger generation at the postsynaptic side. NO can then act retrogradely on presynaptic bouton(s) to activate soluble guanylyl cyclase (sGC) and induce the formation of multiple boutons in contact with one dendritic spine, thus promoting the formation of MIS (Nikonenko et al., 2008). Crucially, recent *in vivo* evidence suggests that MIS generation in the hippocampus may facilitate long-term memory formation (Radwanska et al., 2011; Giese et al., 2015; Aziz et al., 2019). Although findings have begun to decipher the role of MIS on hippocampal-mediated memory, the exact molecular mechanisms that trigger MIS formation during experience-dependent learning and memory have not been fully established.

Wnt secreted proteins are a family of synaptic modulators that play a crucial role in synapse assembly and function in the developing and mature brain. In the hippocampus, Wnts regulate neurotransmitter release at the presynaptic level (Cerpa et al., 2008; Ciani et al., 2015), whereas Wnts at the postsynaptic level increase synaptic NMDAR and α-amino-3-hydroxy-5-methyl-4-isoxazolepropionic acid receptor (AMPAR) levels, dendritic spine growth and synaptic transmission (Cerpa et al., 2011, 2015; Ciani et al., 2011; McQuate et al., 2017; McLeod and Salinas, 2018). Moreover, Wnt expression and/or release is increased by synaptic activity in hippocampal neurons (Chen et al., 2006; Wayman et al., 2006; Gogolla et al., 2009; McLeod et al., 2018). Importantly, LTP induction rapidly increases synaptic Wnt7a/b protein levels which are required for LTP-associated spine growth and synaptic strength (McLeod et al., 2018). Given that Wnt proteins modulate structural and functional plasticity, the Wnt signaling cascade could contribute to activity-mediated generation of MIS.

Here, we investigated whether Wnt signaling contributes to activity-dependent MIS formation. Our results show that LTP induces the formation of MIS through Wnt signaling in hippocampal neurons as LTP-induced MIS formation is blocked by acute blockade of Wnts with a specific Wnt antagonist. Moreover, we show that Wnt7a, which strongly promotes spine growth and is regulated by chemical LTP induction in the hippocampus, acts postsynaptically through the specific Wnt scaffold protein Disheveled 1 (Dvl1) to induce the formation of MIS. Importantly, we demonstrate that Wnt-Dvl1 signaling promotes MIS formation through activation of nNOS at the postsynaptic side and subsequent stimulation of sGC, most likely through NO diffusion to the presynaptic side. Altogether, our results demonstrate for the first time that Wnt signaling acts through NO to retrogradely promote the formation of multiple presynaptic inputs on spines during synaptic plasticity.

## Results

### Chemical LTP Increases the Number of MIS Through Wnts

**FIGURE 1:**
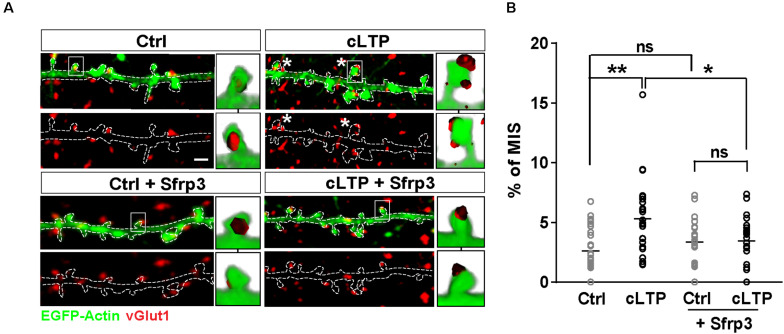
LTP-mediated increase in MIS number requires endogenous Wnts. **(A)** EGFP-actin-expressing cultured neurons (13–14 DIV) (in green) exposed to control (Ctrl) or cLTP conditions, with or without the specific Wnt antagonist Sfrp3 and immunostained for vGlut1 (in red). cLTP induction increased the proportion of spines contacted by more than one vGlut1 puncta (MIS; asterisks). However, this effect was blocked by Sfrp3. Three-dimensional zoomed-in images display front (top) and back (bottom) representations of innervated spines with single or multiple vGlut1 puncta. Scale bar = 1 μm. **(B)** Quantification of mean percentage of MIS in each condition (*n* = 25–31 cells/condition from 3 independent cultures, *P < 0.05 and **P < 0.01 by two-way ANOVA followed by Tukey *post hoc* test).

Previous studies have shown that Wnt signaling promotes synaptic strength and spine growth during LTP (McLeod et al., 2018) and that LTP induction increases the number of MIS (Nikonenko et al., 2003). These findings led us to hypothesize that Wnt signaling could contribute to LTP-mediated formation of MIS in hippocampal neurons. To test this idea, NMDAR-mediated chemical LTP (cLTP) was induced in hippocampal cultures. We found that cLTP increased the number (control = 31.45 ± 0.7 spines/100 μm; cLTP = 44.45 ± 1.3 spines/100 μm; Student’s *t*-test P < 0.001) and width (Control = 0.65 ± 0.01 μm; Glycine = 0.8 ± 0.02 μm; Student’s *t*-test *P* < 0.001) of dendritic spines, in agreement with previous publications (Fortin et al., 2010; McLeod et al., 2018). Under basal conditions, MIS accounted for approximately 2% of all spines (as determined by the number of spines contacted by more than one vGlut1 puncta in three-dimensional (3-D) reconstructions of confocal z-stacks), whereas cLTP induction increased the proportion of MIS to approximately 5% (Figures 1A,B). We next investigated whether Wnts, which are elevated by LTP (Chen et al., 2006; McLeod et al., 2018), contribute to this cLTP-mediated structural plasticity. To block endogenous Wnts, we used the specific Wnt antagonist Sfrp3, a secreted protein that we have previously shown can block the function of Wnts at synapses (Sahores et al., 2010). Exposure to Sfrp3 during cLTP induction completely blocked the increase in MIS number induced by the potentiating stimulus (Figures 1A,B). These results demonstrate that endogenous Wnts are required for activity-induced formation of MIS.

### Wnt7a Promotes the Formation of MIS Through Postsynaptic Activation of Wnt Signaling

**FIGURE 2:**
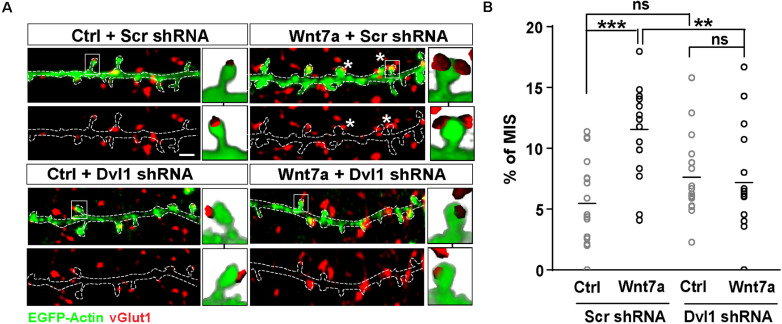
Wnt7a-Dvl1 signaling is required postsynaptically to promote MIS formation. **(A)** Scrambled (Scr) control or Dvl1 shRNA expressing neurons (13–14 DIV) exposed to control (Ctrl) or Wnt7a conditions for 3 h (scale bar: 1 μm). Wnt7a-mediated increase in MIS (asterisks) requires Dvl1. Representative images of EGFP-actin dendrites (in green) and vGlut1 (in red) are shown. Three-dimensional zoomed-in images display front (top) and back (bottom) representations of innervated spines with single or multiple vGlut1 puncta. **(B)** Quantification of mean percentage of MIS in each condition (*n* = 16–18 cells/condition from 3 cultures, **P < 0.01 and ***P < 0.001 by two-way ANOVA followed by Tukey *post hoc* test).

We have recently shown that synaptic activity increases the levels of Wnt7a/b protein at dendritic spines and that Wnt7a signals postsynaptically to regulate LTP-mediated spine growth and AMPAR recruitment (McLeod et al., 2018). We therefore examined if Wnt7a could induce MIS formation. We found that exposure to Wnt7a significantly increased the number of MIS (Figures 2A,B).

To address whether postsynaptic activation of the Wnt pathway was required to promote MIS formation, we specifically blocked Wnt signaling on the postsynaptic side. Wnt7a is known to promote spine growth and synaptic strength through Dvl1 at the postsynaptic compartment (Ciani et al., 2011). Dvl1 is a cytoplasmic scaffold protein that is required for Wnt signaling (Gao and Chen, 2010). Importantly, expression of Dvl1 activates the Wnt signaling pathway in a cell autonomous manner (Boutros and Mlodzik, 1999). Indeed, expression of Dvl1 results in dendritic spine enlargement and increased innervation of spines, with a concomitant increase in mEPSC amplitude and frequency as observed with gain-of-function of Wnt7a (Ciani et al., 2011). We therefore examined whether knock-down of Dvl1 affects Wnt7a-induced formation of MIS.

We first generated two different short hairpin RNA (shRNA) sequences against Dvl1. Dvl1 shRNAs were validated in normal rat kidney (NRK) epithelial cells by quantitative PCR (qPCR), which revealed a 20% reduction in endogenous Dvl1 mRNA levels using clone 1 and a 50% reduction using clone 2 (Supplementary Figure S1). Dvl1 shRNA clone 2 was used for subsequent experiments in primary hippocampal neurons and was co-transfected with EGFP-actin to allow identification and assessment of transfected dendrites contacted by non-transfected axons. We found that loss of postsynaptic Dvl1 function had no significant effect on basal conditions (Figures 2A,B). However, MIS formation was completely abolished following addition of recombinant Wnt7a (Figures 2A,B), suggesting that postsynaptic Wnt signaling is required for Wnt7a-induced MIS formation.

**FIGURE 3:**
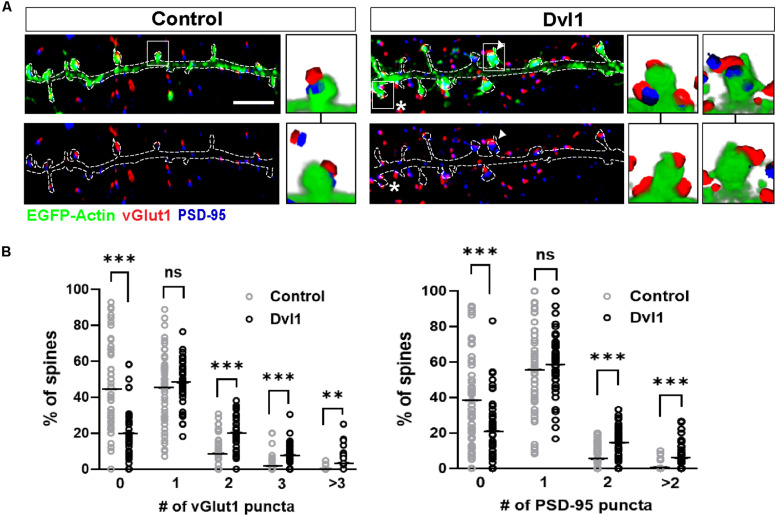
Postsynaptic Dvl1 gain of function increases the innervation of spines and the formation of MIS. **(A)** Representative images of dendrites expressing EGFP-actin (green) and empty vector (control) or Dvl1-HA and immunostained for vGlut1 (red) and PSD-95 (blue). Dvl1 expression increases the proportion of spines contacted by more than one vGlut1 (asterisks) puncta and spines containing more than one discrete PSD-95 puncta (multiple PSDs; arrowhead) when compared to control neurons. Scale bar = 5μm. Three-dimensional zoomed-in images display front (top) and back (bottom) representations of spines with one or more vGlut1 and PSD95 puncta. **(B)** Quantification of the distribution of number of vGlut1 and PSD-95 puncta per spine (*n* = 37–42 cells from 3 independent cultures, **P < 0.01, ***P < 0.001, Mann-Whitney test).

Next, to investigate whether postsynaptic activation of the Wnt pathway enhances the formation of MIS, we performed gain-of-function studies on Dvl1. We therefore expressed Dvl1 in cultured hippocampal neurons co-transfected with EGFP-actin. We found that under control conditions, 45% of innervated spines are contacted by a single vGlut1-labeled presynaptic bouton and 45% are non-innervated. In contrast, expression of Dvl1 decreased the number of non-innervated spines (to 20%), did not affect the number of single innervated spines but increased the proportion of MIS containing two, three or more vGlut1-labeled boutons (Figures 3A,B). The presence of MIS in dendritic spines expressing Dvl1 compared to controls was further demonstrated in 3D movies (Supplementary Movies S1, S2). We also examined the impact of Dvl1 gain-of-function on the number of spines containing the postsynaptic marker PSD-95, which has been shown to modulate MIS formation (Nikonenko et al., 2008). We previously showed that Dvl1 gain-of-function increases the number of spines containing PSD-95 and the volume of PSD-95 per spine (Ciani et al., 2011). Consistently, we found a similar increase in the proportion of spines containing more than one PSD-95 puncta (Figures 3A,B). Our studies demonstrate that even though Dvl1-expressing dendrites were contacted by axons with basal Dvl1 levels, postsynaptic gain- of- function of Dvl1 was able to promote the formation of MIS, implying that the presynaptic side responds to signals from the postsynaptic side by assembling new synaptic boutons.

### Postsynaptic Activation of the Wnt Pathway Requires Nitric Oxide Signaling to Regulate MIS Formation

How does postsynaptic Dvl1 promote the formation of new presynaptic boutons? One possible explanation is that retrograde signals like NO could mediate this effect. The enzyme essential for NO synthesis in neurons, nNOS, is localized at the PSD in dendritic spines of hippocampal neurons (Burette et al., 2001). In contrast, the NO receptor, soluble guanylyl cyclase (sGC), has two isoforms (NO-GC1 and NO-GC2) located at both the pre- and the postsynaptic terminals, with NO-GC1 found predominantly presynaptically (Aoki et al., 1998; Burette et al., 2001, 2002; Neitz et al., 2014). Importantly, MIS formation following postsynaptic expression of PSD-95 or SAP97 is blocked by inhibition of NO signaling, whereas the NO donor DETA increases MIS formation in hippocampal slices (Nikonenko et al., 2008; Poglia et al., 2011). Therefore, we hypothesized that postsynaptic activation of Wnt signaling could utilize retrograde NO signaling to promote the formation of MIS by stimulating the assembly of new synaptic boutons onto spines.

**FIGURE 4:**
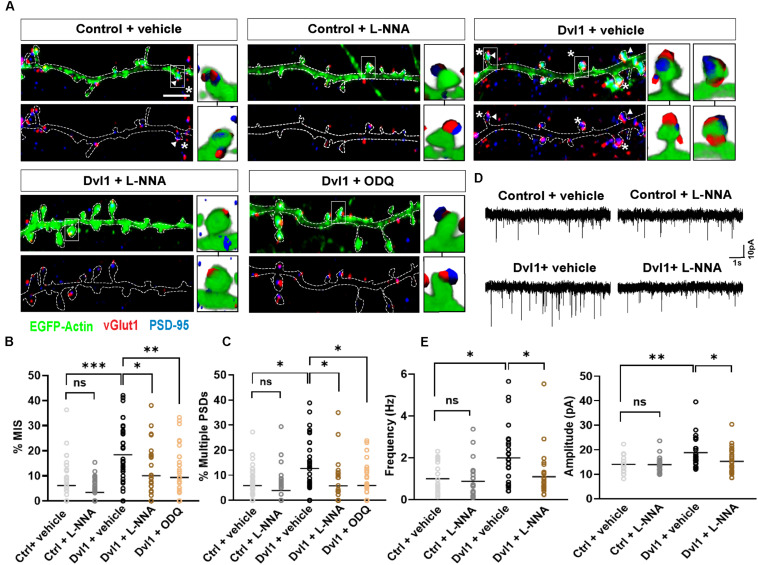
Dvl1 promotes the formation of MIS through NO signaling. **(A)** Representative images of dendrites expressing EGFP-actin (green), or EGFP-actin and Dvl1 and immunostained for vGlut1 (red) and PSD-95 (blue). Following Dvl1 expression, neurons were treated with vehicle, the NOS inhibitor L-NNA or the sGC inhibitor ODQ. NO inhibition blocks the Dvl1 mediated formation of MIS (asterisks) and multiple PSDs (arrowhead). Scale bar = 5 μm. Three-dimensional zoomed-in images display front (top) and back (bottom) representations of spines with one or more vGlut1 and PSD95 puncta. **(B,C)** Quantification of mean percentage of MIS **(B)** and spines **(C)** with multiple PSDs (*n* = 37–42 cells from 3 independent cultures, *P < 0.05, **P < 0.01, ***P < 0.001, n.s. = non-significant by Kruskal-Wallis test followed by Dunn’s *post hoc* test). **(D)** Representative 10 s traces of mEPSCs recorded from neurons, co-transfected with EGFP-actin and empty vector (control) or Dvl1-HA and treated with vehicle or the NOS inhibitor L-NNA. Dvl1 increases mEPSC frequency and amplitude, both of which are blocked by L-NNA. **(E)** Quantification of mean mEPSC frequency and amplitude [*n* = 21–29 cells/condition from 3 independent cultures *P < 0.05, **P < 0.01 by Kruskal-Wallis test followed by Dunn’s *post hoc* test in (frequency) and two-way ANOVA followed by Tukey *post hoc* test in (amplitude)].

To assess this, we applied the nNOS inhibitor NG-nitro-L-arginine (L-NNA) or the sGC inhibitor 1H-[1,2,4] oxadiazolo[4,3-a]quinoxalin-1-one (ODQ) onto control EGFP-actin expressing neurons or neurons co-expressing EGFP-actin and Dvl1. Under basal conditions, inhibition of nNOS by L-NNA had no effect on MIS formation and on spines with multiple PSDs (Figures 4A–C). As described above (Figure 3), Dvl1 gain-of-function increased the percentage of MIS and spines with multiple PSDs (Figures 4A–C). Crucially, inhibition of NO-sGC signaling by either L-NNA or ODQ blocked the effect of Dvl1 on both the number of MIS and multiple PSDs (Figures 4A–C). Importantly, inhibition of nNOS did not affect spine number and size under basal conditions or block Dvl1-induced spine enlargement (Supplementary Figure S2A). Similarly, inhibition of sGC by ODQ did not affect Dvl1-associated spine size enhancement (Supplementary Figure S2A). In contrast, NO-sGC signaling inhibition blocked Dvl1-mediated increase in the percentage of excitatory synapses as determined by colocalization of vGlut1 and PSD-95 (Supplementary Figure S2B). Moreover, L-NNA also reduced the number of excitatory synapses (Supplementary Figure S2B) under basal conditions suggesting that NO signaling plays a role in excitatory synapse formation in hippocampal neurons. Collectively, these results demonstrate that activation of postsynaptic Wnt signaling promotes MIS formation and synaptic connectivity through NO-sGC signaling.

Given the profound effect of NO-sGC signaling blockade on Wnt mediated MIS formation, we investigated the impact of this pathway at the functional level by performing electrophysiological recordings. Previous studies have shown that Dvl1-induced changes in spine innervation and morphology have functional correlates, as the frequency and amplitude of miniature excitatory postsynaptic currents (mEPSC) are increased by gain of function of Dvl1 on the postsynaptic side (Ciani et al., 2011). We therefore tested whether inhibition of NO signaling blocks these synaptic functional changes. Under control conditions in primary hippocampal neurons (Supplementary Figure S2B), L-NNA did not affect either the frequency or amplitude of mEPSCs suggesting preserved basal transmission properties with inhibition of NO production (Figures 4D,E). In contrast, L-NNA completely blocked the ability of Dvl1 to increase mEPSC frequency and amplitude (Figures 4D,E), suggesting that NO signaling is required for Dvl1-mediated functional changes at excitatory synapses.

## Discussion

Neuronal activity induces long lasting structural modifications in synapses to promote changes in synaptic strength. For example, LTP induction results in the enlargement of dendritic spines and the formation of multi-contact synapses (Hruska et al., 2018; Zaccard et al., 2020), including MIS, a unique form of structural synaptic plasticity. Indeed, formation of MIS is associated with NMDAR-dependent LTP (Nikonenko et al., 2003; Hruska et al., 2018). Here, we have uncovered a novel role for Wnt signaling in MIS generation. We demonstrate that enhanced MIS formation during NMDAR-mediated cLTP requires Wnt signaling. Specifically, Wnt7a, which is expressed in the hippocampus, promotes the formation of MIS through postsynaptic activation of the Wnt pathway. This results in activation of NO signaling and a retrograde modulation of presynaptic inputs and mEPSC frequency and amplitude. Our findings unravel a molecular mechanism by which Wnt signaling acts through the NO-sGC cascade to trigger activity-induced MIS formation.

Wnts are required for the formation of MIS during NMDAR-mediated cLTP in hippocampal neurons. Previous studies have demonstrated that LTP-dependent activation of NMDAR increases MIS number in hippocampal organotypic cultures (Nikonenko et al., 2003). However, the initial signal(s) that triggers LTP-mediated MIS formation has not been demonstrated. We focused our attention on Wnts for several reasons. First, Wnt proteins are required for synapse formation, spine enlargement and synaptic strengthening (Sahores et al., 2010; Ciani et al., 2011; McLeod et al., 2018). Second, activation of Wnt signaling postsynaptically increases the content of PSD-95 in dendritic spines (Ciani et al., 2011). Third, we have shown that endogenous Wnt7a/b levels are elevated upon induction of LTP and that Wnt7a signals postsynaptically to regulate LTP-mediated spine growth and AMPAR recruitment (McLeod et al., 2018). Here we demonstrate that Wnt7a also increases MIS formation and the number of spines with multiple PSD-95 puncta. These results are consistent with the finding that PSD-95 is required for dendritic spine stabilization during LTP (Stein et al., 2003; Ehrlich et al., 2007) and MIS formation (Nikonenko et al., 2008; Radwanska et al., 2011). To date, no studies have examined how stable these structural changes are over time. Nonetheless, together our results suggest that the elevation of Wnt7a protein following LTP induction is important for dynamic pre- and postsynaptic structural plasticity.

Specific activation of the Wnt pathway on the postsynaptic side promotes MIS formation. Previous studies have shown that gain of function of Dvl1, which is enriched at dendritic spines in hippocampal neurons, can activate the Wnt pathway in a cell autonomous manner (Ciani et al., 2011). Notably, we found that Dvl1 gain-of-function on the postsynaptic side is sufficient to induce MIS formation. Conversely, postsynaptic loss-of-function of Dvl1 blocks the ability of Wnt7a to promote MIS formation. These results suggest that activation of the Wnt pathway on the postsynaptic dendrite/spine triggers a signal that acts retrogradely to promote the assembly of several synaptic boutons on a single spine.

Consistent with the generation of a retrograde signal, our studies identify NO as the retrograde messenger required for Wnt-induced MIS formation. Under basal conditions, inhibition of nNOS (located postsynaptically) had no effect on MIS formation or on the number of spines with multiple PSDs. In contrast, postsynaptic gain of function of Dvl1, that activates the Wnt pathway in a cell autonomous manner on the postsynaptic side, promoted MIS formation, an effect that was blocked by NO-sGC inhibition. Based on these results, we propose that activation of the Wnt pathway on the postsynaptic side triggers NO synthesis through nNOS. NO then diffuses retrogradely to the presynaptic site to promote the formation of several boutons innervating a single spine through sGC activation. This effect is likely mediated through the NO-GC1 isoform, which is predominantly located on the presynaptic terminal (Neitz et al., 2014). These findings are in agreement with previous studies showing that MIS formation requires NO signaling (Nikonenko et al., 2003, 2008; Poglia et al., 2011). Indeed, expression of PSD-95 is sufficient to promote MIS formation through production of NO (Nikonenko et al., 2008). Therefore, it is reasonable to assume Wnt7a through Dvl1 increases PSD-95 levels in spines promoting MIS formation through nNOS/NO/sGC signaling. Importantly, these structural modifications are accompanied by an increase in mEPSC frequency with no changes in spine number, suggesting a functional correlate in response to MIS formation. Interestingly, increasing evidence indicates that Wnts modulate NO signaling in several contexts. For example, Wnt5a regulates NMDAR trafficking and potassium voltage-gated currents through NO production in the hippocampus (Munoz et al., 2014; Parodi et al., 2015). Our studies also highlight a key role for NO production in hippocampal excitatory synapse formation without affecting mEPSC frequency under basal conditions. Similar findings are observed when blockade of NO production occurs in hippocampal organotypic slices (Nikonenko et al., 2013). Overall, these results demonstrate a dynamic interplay between multiple Wnts and NO under basal and synaptic plasticity conditions to regulate pre- and postsynaptic function.

What is the cognitive impact of MIS formation? MIS generation has been associated with learning and long-term memory. Indeed, complex motor learning increases MIS number in layer II/III of the motor cortex (Jones et al., 1999). Other memory tasks, including auditory fear conditioning, also lead to MIS generation in the auditory cortex (Yang et al., 2018). Recent *in vivo* studies have demonstrated that MIS formation is increased at CA1 hippocampal neurons after contextual fear memory formation (Aziz et al., 2019). These findings suggest that MIS generation is an efficient mechanism to increase connectivity and/or strengthen connections on existing synapses during learning. Furthermore, in neurological disorders characterized by increased cortical connectivity and disrupted network function such as Fragile X syndrome (Bureau et al., 2008; Zhang et al., 2014), the number of MIS are also significantly increased (Booker et al., 2019). This structural plasticity mechanism could be driven by differences in cortical network activity. Interestingly, experience-driven neuronal activity induces structural changes at the synapse through Wnts. Environmental enrichment causes Wnt7a/b upregulation in the CA3 hippocampal region, which promotes the formation of multiple mossy fiber terminals and increases the density of synapses per mossy fiber terminal (Gogolla et al., 2009). Furthermore, *in vivo* studies show that Wnt7a loss of function delays the maturation of glomerular rosettes, complex multisynaptic structures formed between a mossy fiber axon and dendrites from numerous granule cells in the cerebellum (Hall et al., 2000). *In vivo*, structural synaptic plasticity, LTP and memory formation also require Wnts (Marzo et al., 2016). Thus, Wnt signaling is required for activity- or experience-mediated formation of complex synaptic structures. Here we uncover a novel role for Wnt7a-Dvl1 signaling in LTP-mediated MIS formation. We demonstrate that the Wnt cascade modulates NO signaling to regulate structural plasticity at both sides of the synapse in a coordinated manner thus contributing to increased synaptic connectivity.

## Materials and Methods

### Hippocampal Cultures, Cell Transfection, and Treatments

Primary hippocampal neurons (250 cells/mm^2^) were isolated from embryonic day 18 Sprague-Dawley rat embryos and cultured as previously described (Dotti et al., 1988). To overexpress Dvl1, cultures were co-transfected at 7-8 DIV with EGFP-actin and Dvl1-HA or empty vector (PCS2) using calcium phosphate. To knockdown Dvl1, cultures were co-transfected using calcium phosphate with EGFP-actin (0.5 μg) and scrambled or Dvl1 shRNA (0.05 μg). Scrambled (5′-GGCGTTACGTCCTAACATGCG-3′) and two Dvl1 shRNA (clone #1: 5′-GGGTCTAACTTACTTATTTAT-3′; clone #2: 5′-CTTGAATCTAGCAGCTTTATT-3′) target sequences were cloned into an AAV-U6 vector expressing mCherry. Dvl1 shRNA clone #2 was used for experiments. Purified recombinant Wnt7a (150 ng/mL; PeproTech) was applied to neurons at 37°C for 3 h. Bovine serum albumin (BSA) was used as a control. 100 μM L-NNA or 10 μM ODQ were added to cultures at 9 DIV and re-added at 11 DIV. Vehicles for L-NNA and ODQ were equimolar HCl and DMSO, respectively, as previously described (Bartus et al., 2013). After appropriate treatment, 12 DIV neurons were fixed for immunofluorescence or used for electrophysiological experiments.

### Real-Time PCR

NRK cells were transfected with scrambled or Dvl1 shRNA clones as described above using electroporation and Nucleofector (Lonza). After 24 h, cells were washed in cold PBS and homogenized using Trizol Reagent (Life Technologies). Total RNA was then extracted using Direct-zol columns (ZymoResearch). RNA concentration was quantified using a NanoDrop ND-100 (Thermo Scientific). Up to 2000 ng of RNA were used for cDNA synthesis using RevertAid H Minus First Strand cDNA Synthesis kit (Themo Fisher Scientific). Five nanogram of original RNA was used to perform fast qPCR using GoTaq qPCR Master Mix (Promega) in a LigherCycler^®^ 480 (Roche). Primers for Dvl1 and three housekeeping genes (Gapdh, Actb and Rps18) were designed using OligoPerfect design (Thermo Fisher Scientific) and validated using *in silico* PCR (UCSC genome Browser) and Ensembl BLAST^1^. Primers (*Dvl1* Fw: 5′-GCTGAAGCATGGTTTCCTGC-3′; *Dvl1* Rv: 5′-GTTGAGGTTCAGGGATGCGA-3′; *Actb* Fw 5′-GGCTCCTAGCACCATGAAGA-3′; *Actb* Rv: 5′- CTGGAA GGTGGACAGTGAGG-3′; *Gapdh* Fw 5′-AGACAGCCGCATC TTCTTGT-3′; *Gapdh* Rv: 5′-CTTGCCGTGGGTAGAGTCAT-3′; *Rps18* Fw 5′-CTTCCACAGGAGGCCTACAC-3′; *Rps18* Rv: 5′-GTACTCGCAGGATGTGCTGA-3′) were used at 0.5 μM (Sigma Aldrich).

### Chemical Long-Term Potentiation (cLTP)

LTP was induced in 13–14 DIV hippocampal cultures using an NMDAR mediated chemical LTP (cLTP) protocol as previously described (Fortin et al., 2010; McLeod et al., 2018). Briefly, hippocampal neurons were kept at room temperature (RT) for 20–30 min in control solution (125 mM NaCl, 2.5 mM KCl, 1 mM MgCl_2_, 2 mM CaCl_2_, 33 mM D-glucose, 5 mM HEPES, 20 μM D-APV, 3 μM strychnine, 20 μM bicuculline and 0.5 μM TTX; pH 7.4). After cLTP induction (addition of glycine 200 μM for 10 min in the absence of Mg^2+^, D-APV and TTX) cultures were returned to control solution for 1 h prior to fixation (all performed at RT). To block endogenous Wnt proteins, recombinant Sfrp3 (250 ng/mL; R&D Systems) was used only throughout the induction of cLTP and after.

### Immunofluorescence, Image Acquisition, and Analyses

Dissociated neurons were fixed with cold 100% methanol or 4% paraformaldehyde (PFA)/4% sucrose in PBS for 20 min at RT, permeabilized with 0.025% Triton, blocked with 5% BSA and then incubated with primary antibodies overnight at 4°C. Primary antibodies against GFP (1:500; Millipore), vGlut1 (1:10,000; Chemicon) and PSD-95 (1:500; Affinity Bioreagents) were used. Secondary antibodies were Alexa 488, 568, and 647 (1:600 dilution, Molecular Probes). Fluorescence images of pyramidal neurons were captured with a Leica TCS SP1 Confocal microscope using a 63x oil objective (NA = 1.32), producing image stacks of 157.8 × 157.8 μm with an average z-depth of ∼5 μm. Six to twelve images were taken per condition per experiment and analyzed blind to the experimental condition using Volocity (Improvision). For each EGFP-actin-transfected cell, 2–3 regions of interest containing ∼50–100 μm of secondary dendrite were cropped from maximum projections. The number of spines were counted, and the spine head/size width determined with a line tool. Finally, the number of spines containing PSD-95, vGlut1 and multiple PSD-95 and/or vGlut1 puncta was counted. The 3D visualization tool on Volocity was used to confirm if synaptic puncta were in the same focal plane as spines. MIS were defined as EGFP-actin spines contacted by more than one vGlut1 puncta. To ensure our MIS quantification did not include vGlut1 that could feasibly come from the same presynaptic terminal, we excluded vGlut1 within ∼0.2–0.3 μm of each other. For each condition, approximately 1000 spines were analyzed in total from all three repeats of each experiment.

The 3D images and movies (.avi files) of individual spines were obtained from stacks of confocal images (as above), cropped and then processed using the animation function in Imaris software to zoom and navigate around the spines.

### Electrophysiology

Whole-cell patch clamp recordings were performed on primary hippocampal neuron cultures co-transfected with EGFP-actin (1 μg) and Dvl1-HA (1 μg) or empty vector. Most neurons co-express EGFP-actin and Dvl1-HA. Coverslips were placed on an upright microscope and continuously perfused at RT with recording solution containing (in mM): NaCl (125), NaHCO_3_ (25), KCl (2.5), NaHPO_4_ (1.25), CaCl_2_ (1), MgCl_2_ (1), D-glucose (25) bicuculine (0.01), and TTX (0.0001). Cells were voltage-clamped at -60 mV in the whole cell configuration using borosilicate glass microelectrodes (resistance 5–8 MΩ) filled with a pipette solution containing (in mM): D-gluconic acid lactone (139), HEPES (10), EGTA (10), NaCl (10), CaCl_2_ (0.5), MgCl_2_ (1), ATP (1) and GTP (1) adjusted to pH 7.2 with CsOH. mEPSCs were recorded using an Axopatch 200B amplifier, filtered at 1 kHz and digitized at 10 kHz using WinEDR. Currents were analyzed blind using a combination of WinEDR and WinWCP (freely available at http://spider.science.strath.ac.uk/sipbs/software_ses.htm).

### Statistical Analyses

All data are represented as mean ± SEM from at least three independent experiments (unless otherwise stated). Statistical analyses were performed on GraphPrism and Origin with data normality assessed using Kolmogorov-Smirnov tests. Normally distributed data were analyzed using an unpaired Student’s *t*-test, one-way or two-way ANOVA with Tukey’s *post hoc* correction for multiple comparisons. Mann-Whitney and Kruskal-Wallis tests with Dunn’s post-test were used for non-parametric data.

## Data Availability Statement

The raw data supporting the conclusions of this article will be made available by the authors, without undue reservation, to any qualified researcher.

## Ethics Statement

The animal study was reviewed and approved by the UCL Animal Welfare and Ethical Review Body (Bloomsbury Campus).

## Author Contributions

PS conceived the overall project, guided the project, and provided the funding. AG contributed to the design and analyses of the electrophysiology experiments. FM and AM performed the cell biology experiments. KB performed the cell biology and electrophysiology experiments. NM-F evaluated the results. TM evaluated the results presented in Figure 1. EP performed the qPCR experiments. All authors participated in the design of experiments, interpretation of data, and in the writing of the manuscript.

## Conflict of Interest

The authors declare that the research was conducted in the absence of any commercial or financial relationships that could be construed as a potential conflict of interest.

## Footnotes

1 http://ensemble.org/

## Supplementary Material

FIGURE S1 Validation of Dvl1 shRNA knockdown constructs. Dvl1 mRNA levels in NRK cells transfected with scrambled or two different Dvl1 shRNA clones were evaluated. Graph represents fold change in mRNA levels relative to scrambled shRNA control. Dvl1 shRNA clone #2 was used for experiments (*n* = 4 independent cultures, ^∗∗^*P* < 0.01 by One-way ANOVA). Related to Figure 2.

FIGURE S2 Postsynaptic Dvl1-mediated increase in excitatory innervation but not spine size is blocked by inhibition of NO signaling. **(A)** Quantification shows that Dvl1 does not affect spine number and this is not affected by inhibition of NO signaling. In addition, the effect of Dvl1 on increased spine head width is unaffected by inhibition of NO signaling. **(B)** Quantification shows that inhibition of NO signaling blocks the effect of Dvl1 on the proportion of spines contacted by vGlut1 puncta, containing PSD-95 puncta, or both. L-NNA also reduces basal innervation (*n* = 37–42 cells from 3 independent experiments, ^∗^*P* < 0.05, ^∗∗^*P* < 0.01, ^∗∗∗^*P* < 0.001, n.s. = non-significant, Kruskal-Wallis test followed by Dunn’s *post hoc* test). Related to Figure 3.

MOVIE S1 Single innervation of a spine in a control neuron expressing EGFP-actin (green). Representative 3D reconstruction of a dendritic spine where presynaptic boutons are labeled with vGlut1 (red) and the PSD with PSD-95 (blue). Related to Figure 3.

MOVIE S2 Postsynaptic Dvl1 expression increases the number of MIS. Representative 3D reconstruction of a dendritic spine expressing EGFP-actin (green) and Dvl1-HA. Presynaptic boutons and PSD were labeled with vGlut1 (red) and PSD-95 (blue) respectively. Related to Figure 3.
