# Supplementary figures and images for: Wnt Signaling Through Nitric Oxide Synthase Promotes the Formation of Multi-Innervated Spines
Source: Front Synaptic Neurosci. 2020 Sep 4;12:575863. doi: 10.3389/fnsyn.2020.575863 (PMC7509412; doi:10.3389/fnsyn.2020.575863)

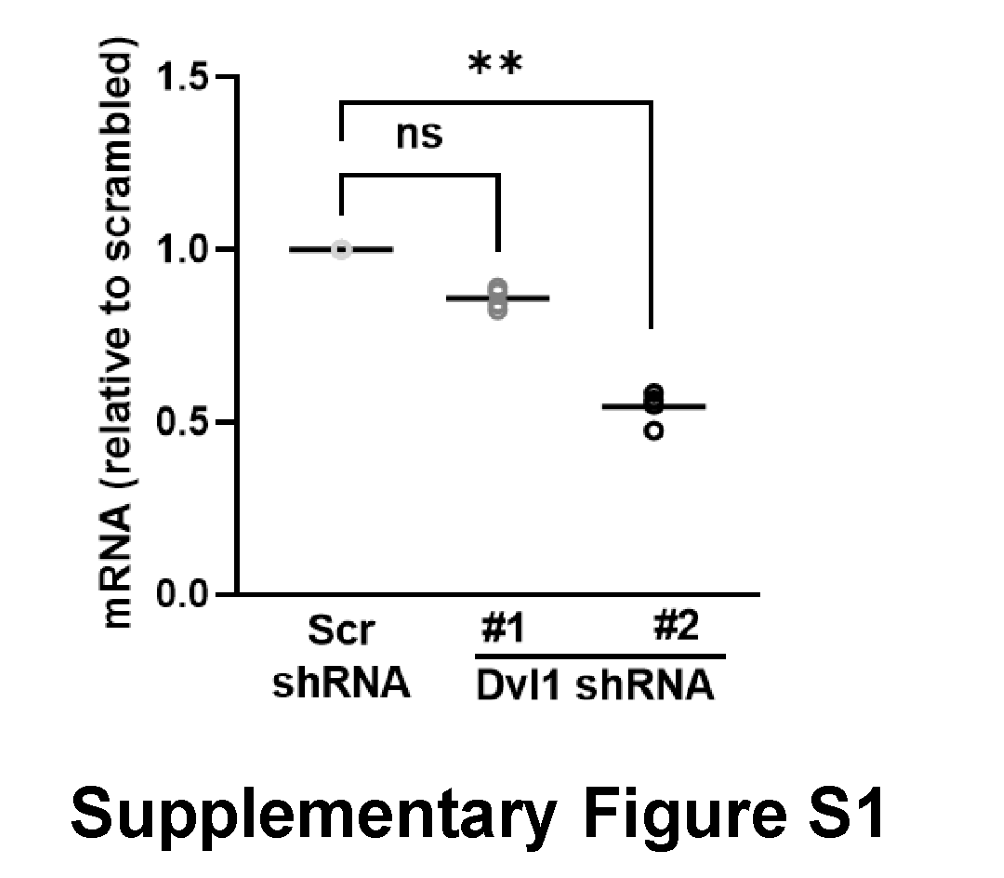

Supplement: FIGURE S1 — Validation of Dvl1 shRNA knockdown constructs. Dvl1 mRNA levels in NRK cells transfected with scrambled or two different Dvl1 shRNA clones were evaluated. Graph represents fold change in mRNA levels relative to scrambled shRNA control. Dvl1 shRNA clone #2 was used for experiments (n = 4 independent cultures, ∗∗P < 0.01 by One-way ANOVA). Related to Figure 2. [file Image_1.TIF]

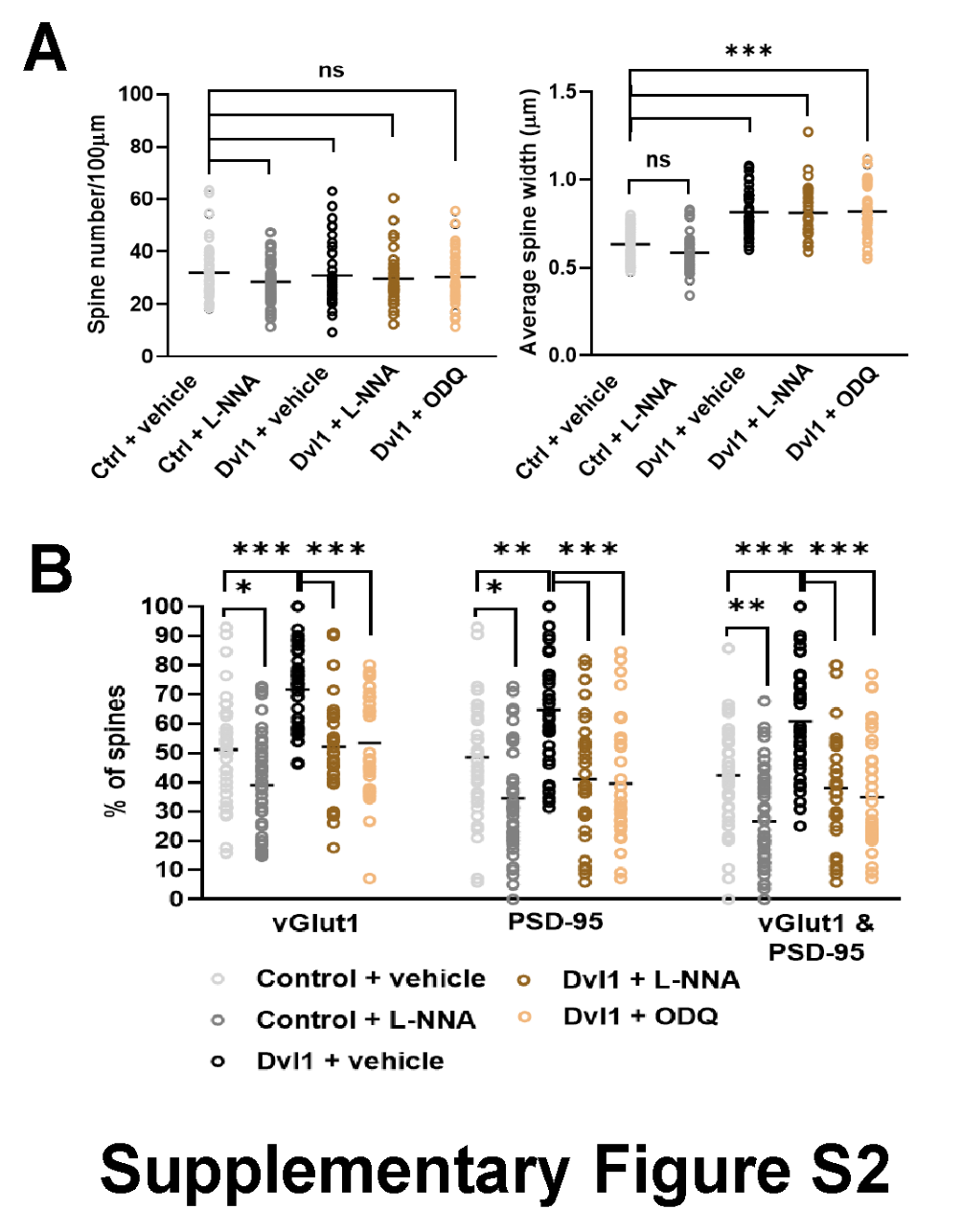

Supplement: FIGURE S2 — Postsynaptic Dvl1-mediated increase in excitatory innervation but not spine size is blocked by inhibition of NO signaling. (A) Quantification shows that Dvl1 does not affect spine number and this is not affected by inhibition of NO signaling. In addition, the effect of Dvl1 on increased spine head width is unaffected by inhibition of NO signaling. (B) Quantification shows that inhibition of NO signaling blocks the effect of Dvl1 on the proportion of spines contacted by vGlut1 puncta, containing PSD-95 puncta, or both. L-NNA also reduces basal innervation (n = 37–42 cells from 3 independent experiments, ∗P < 0.05, ∗∗P < 0.01, ∗∗∗P < 0.001, n.s. = non-significant, Kruskal-Wallis test followed by Dunn’s post hoc test). Related to Figure 3. [file Image_2.TIF]
